# Relationships of serum CC16 levels with smoking status and lung function in COPD

**DOI:** 10.1186/s12931-022-02158-8

**Published:** 2022-09-16

**Authors:** Kelli C. Gribben, Jill A. Poole, Amy J. Nelson, Paraskevi A. Farazi, Christopher S. Wichman, Art J. Heires, Debra J. Romberger, Tricia D. LeVan

**Affiliations:** 1 Department of Epidemiology, University of Nebraska Medical Center, Omaha, NE 68198 USA; 2 Division of Allergy and Immunology, Department of Internal Medicine, University of Nebraska Medical Center, Omaha, NE 68198 USA; 3 Department of Biostatistics, University of Nebraska Medical Center, Omaha, NE 68198 USA; 4 Division of Pulmonary, Critical Care and Sleep, Department of Internal Medicine, University of Nebraska Medical Center, Omaha, NE 68198 USA; 5 VA Nebraska Western Iowa Healthcare System, Omaha, NE 68105 USA

**Keywords:** COPD, CC16, Lung function, Former smokers

## Abstract

**Background:**

The club cell secretory protein (CC16) has anti-inflammatory and antioxidant effects, and low CC16 serum levels have been associated with both risk and progression of COPD, yet the interaction between smoking and CC16 on lung function outcomes remains unknown.

**Methods:**

Utilizing cross-sectional data on United States veterans, CC16 serum concentrations were measured by ELISA and log transformed for analyses. Spirometry was conducted and COPD status was defined by post-bronchodilator FEV_1_/FVC ratio < 0.7. Smoking measures were self-reported on questionnaire. Multivariable logistic and linear regression were employed to examine associations between CC16 levels and COPD, and lung function with adjustment for covariates. Unadjusted Pearson correlations described relationships between CC16 level and lung function measures, pack-years smoked, and years since smoking cessation.

**Results:**

The study population (N = 351) was mostly male, white, with an average age over 60 years. An interaction between CC16 and smoking status on FEV_1_/FVC ratio was demonstrated among subjects with COPD (N = 245, p = 0.01). There was a positive correlation among former smokers and negative correlation among current or never smokers with COPD. Among former smokers with COPD, CC16 levels were also positively correlated with years since smoking cessation, and inversely related with pack-years smoked. Increasing CC16 levels were associated with lower odds of COPD (OR_adj_ = 0.36, 95% CI 0.22–0.57, P_adj_ < 0.0001).

**Conclusions:**

Smoking status is an important effect modifier of CC16 relationships with lung function. Increasing serum CC16 corresponded to increases in FEV_1_/FVC ratio in former smokers with COPD versus opposite relationships in current or never smokers. Additional longitudinal studies may be warranted to assess relationship of CC16 with smoking cessation on lung function among subjects with COPD.

**Supplementary Information:**

The online version contains supplementary material available at 10.1186/s12931-022-02158-8.

## Background

Chronic obstructive pulmonary disease (COPD) is a significant public health problem affecting an estimated 384 million (11.7%) globally, and is a leading cause of death [1, 2]. COPD is an obstructive airway disease characterized by chronic inflammation, an altered inflammatory response to respiratory insults such as cigarette smoke, and structural changes eventually leading to air trapping and decreased lung function [1]. Smoking is the primary environmental risk factor for developing COPD, although several other exposures can also contribute to disease risk such as air pollution and occupational exposures. The Club Cell Secretory Protein (CC16) is a pneumoprotein with anti-inflammatory and antioxidant properties, and is an emerging biomarker for COPD [3–5]. Low baseline serum CC16 levels have been associated with lung function deficits [6, 7], and incident COPD in two large prospective cohorts [6]. A similar relationship was observed in a meta-analysis of over 1,100 adolescents from three birth cohorts, where low serum CC16 measured at ages 4–6 years was associated with a 68 mL deficit in FEV_1_ measured at age 8–16 years [6]. There is also interest in exploring the potential therapeutic benefits of supplementing CC16 in COPD patients based upon preliminary experimental evidence [8–10].

CC16 is almost exclusively produced by club cells and other non-ciliated airway epithelial cells, making up 7% of all proteins in bronchoalveolar lavage fluid (BALF) [4]. CC16 moves from the airway into circulation via passive transportation upon injury to the epithelium from inhaled toxins such as cigarette smoke [11]. Acute exposure to cigarette smoke temporarily increases circulating CC16, while chronic exposure to cigarette smoke is associated with decreased levels in serum and BALF [4, 11]. In addition, it has been consistently reported that serum levels are lower in subjects with COPD compared to controls [4, 12–14]. Furthermore, in COPD, current smokers have lower CC16 levels than former and never smokers [13–15]. Among adults with COPD, lower serum CC16 levels have been associated with lung function decline indicating accelerated disease progression [15, 16].

Although smoking history (pack-years) and smoking status have been recognized as potential confounders in analyses of CC16 and lung function, there remains knowledge gaps in COPD populations. Namely, the relationships between CC16 and smoking status and smoking history, CC16 and lung function measures, and the possible interaction between CC16 and smoking status on lung function outcomes has not been fully described. Moreover, the previously described protective effect of CC16 on risk of COPD requires further validation. Therefore, the aims of this study were to (1) examine relationships between serum CC16 levels and COPD, pack-years smoked, years since smoking cessation, and lung function measures among adults with COPD, and (2) determine if there is an interaction between CC16 level and smoking status related to lung function outcomes in adults with COPD.

## Methods

### Study population

The present study utilized data from a cross-sectional study designed to investigate agricultural exposures and chronic respiratory diseases among United States veterans. The study population has been described in more detail elsewhere [17] but in brief, veterans visiting a primary care outpatient clinic at the VA Nebraska Western Iowa Health Care System between March 2008 and December 2013 were eligible for the study if they had at least 2 years of work experience on a farm as an adult, and were between the ages 40–80 years. Veterans were excluded if they had been diagnosed with asthma, lung cancer or interstitial lung disease. Data collection has been described elsewhere [18] and briefly, demographic and clinical information was obtained from all participants at enrollment and included a questionnaire, blood sample, and spirometry testing to assess lung function. COPD was defined according to the Global Initiative for Chronic Obstructive Lung Disease (GOLD) standard criteria as a post-bronchodilator ratio of forced expiratory volume released in 1 s (FEV_1_) to forced vital capacity (FVC) < 0.70 [1]. COPD subjects were recruited at a stable state (i.e. no history of exacerbations or infection in the previous 3 weeks before enrollment). Controls were participants without COPD (i.e. pre-bronchodilator FEV_1_/FVC ≥ 0.70) that had never smoked. COPD stage was defined by the GOLD criteria (stage I–IV) [1]. The analytical sample included 351 participants, including 245 COPD and 106 control subjects (never smokers). This study was approved by the VA Nebraska Western Iowa Healthcare Systems Institutional Review board and a signed, written informed consent document was collected from all participants.

### CC16

CC16 protein concentration was measured in serum from stored samples using commercially available enzyme-linked immunosorbent assay (ELISA) kits from Bio Vendor (Asheville, NC; lower limit of detection 46 pg/mL). The distribution of serum CC16 levels were right skewed, and therefore levels were log transformed for analysis to approximate a normal distribution.

### Lung function measures

Lung function was evaluated by spirometry in all participants. Post-bronchodilator spirometry was performed if the ratio FEV_1_/FVC was < 0.70 (two doses of 0.083% albuterol) [18]. FEV_1_ and FVC values were analyzed as percentage of predicted value for specific sex, age, height, weight (FVC only) and race according to NHANES III reference equations [19].

### Other variable definitions

Smoking status was defined as current, former, or never using the question, “Have you smoked a total of 100 cigarettes or more during your lifetime?”. Current smokers were defined by the response, “Yes, and I still smoke”, former smokers responded, “Yes, but I no longer smoke”, and never smokers answered, “No, I never smoked.” Pack-years smoked was calculated for current and former smokers. For current smokers, we used the equation (number of cigarettes smoked per day/20 cigarettes per pack) × (current age − age first started smoking regularly), and the following equation for former smokers (number of cigarettes smoked per day/20 cigarettes per pack) × (age last smoked regularly − age first started smoking regularly). Among former smokers, the variable ‘time since quit smoking’ was defined by taking the difference between age last smoked regularly and current age, both self-reported on the questionnaire. Therefore, the ‘time since last smoked’ regularly (at least 3 cigarettes/week for at least 6 months or more) measurement provides an estimate of the duration of smoking cessation (in years).

### Statistical analysis

Descriptive statistics were used to summarize demographic and clinical characteristics of the study population. Visualizations display the median and interquartile range for serum CC16 levels by COPD status, smoking status, and GOLD stage. Student’s t-test and ANOVA were used to evaluate associations between the log CC16 level and COPD, and GOLD stage. Linear regression was employed to assess trends in log CC16 level by smoking status (never, former, current). Covariates included the following: age (years, continuous), body mass index (BMI) kg/m^2^ (continuous), smoking status (never, former, current), pack-years (current and former smokers), assay plate number (categorical), and systemic (i.e. prednisone) or inhaled steroid use in the past 12 months. Pearson correlations were used to examine relationships between log CC16 and each lung function measure. Logistic regression was used to evaluate relationships between COPD and CC16 levels with controls being never smokers. Linear regression models were fit to assess relationships between log CC16 level, and lung function measures adjusted for covariates. A multiplicative interaction term between log CC16 level and smoking status was evaluated in each lung function model. An alternative analysis was performed treating smoking status as a confounder in the model estimating the odds of COPD. A p-value < 0.05 was considered statistically significant. SAS 9.4 was used for all analyses.

## Results

Demographic and clinical characteristics of the study population stratified by COPD status are described in Table 1. COPD subjects were slightly older and less educated than control subjects (never smokers). Both groups were predominately white, male, with an average age over 60 years. Among cases, 30.2% were current smokers, 61.2% former smokers, and 8.7% never smokers. Additionally, more than half (53.3%) were COPD GOLD stage II, and only 6.2% had advanced stage disease (GOLD stage IV), 18.4% took a prescribed inhaled steroid, and 9.8% took systemic steroids (i.e. prednisone) in the past year.

**Table 1:** Characteristics of study population stratified by COPD vs controls (never smokers) N = 351

	COPD N = 245	Control subjects (never smokers) N = 106	p
Age (years), mean (SD)	66.5 (8.3)	62.7 (9.5)	< 0.001
Sex, N (%)			
Male	240 (99.2%)	102 (96.2%)	0.07
Female	2 (0.8%)	4 (3.8%)	
Race, N (%)			
White	234 (97.1%)	103 (97.2%)	0.97
Other	7 (2.9%)	3 (2.8%)	
Education, N (%)			
≤ High school	123 (52.1%)	34 (32.7%)	0.001
> High school	113 (47.9%)	70 (67.3%)	
BMI (kg/m^2^), median (IQR)	29.0 (25.4–33.5)	32.5 (28.2–35.7)	< 0.0001
Smoking status, N (%)			
Current	73 (30.2%)	–	
Former	148 (61.2%)	–	
Never	21 (8.7%)	106 (100.0%)	
Pack-years^a^, median (IQR)	34.0 (13.0–56.0)	0 (0.0)	–
FEV_1_ (% predicted), mean (SD)	65.4 (20.5)	89.8 (16.2)	< .0001
FVC (% predicted), mean (SD)	83.0 (18.7)	86.7 (15.5)	0.07
FEV_1_/FVC, mean (SD)	57.7 (11.0)	77.9 (4.7)	< .0001
COPD GOLD stage, N (%)			
I	62 (25.6%)	–	
II	129 (53.3%)	–	
III	36 (14.9%)	–	
IV	15 (6.2%)	–	

FEV_1_: forced expiratory volume in 1 s; FVC: forced vital capacity; FEV_1_/FVC: the ratio of FEV_1_ to FVC; COPD: chronic obstructive pulmonary disease defined as FEV_1_/FVC < 0.7; COPD GOLD stage: chronic obstructive pulmonary disease global initiative for chronic obstructive lung disease stage; BMI: body mass index (kg/m^2^); IQR: interquartile range = (25th percentile–75th percentile); SD: standard deviation

^a^Pack-years smoked calculated among former and current smokers only

**Fig. 1:**
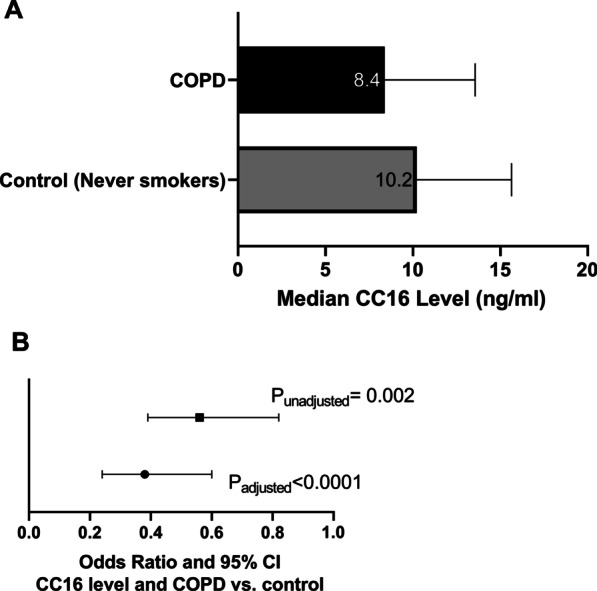
Serum CC16 levels are inversely associated with COPD. **A** Bar graph depicts the median and interquartile range (IQR) of serum CC16 levels (ng/mL) by COPD status (N = 351). Cases had significantly lower levels of CC16 as compared to controls (never smokers) (t-test using log CC16, p = 0.002). **B** Odds ratio plot displays the unadjusted and model-adjusted association (odds ratios and 95% confidence intervals) between log CC16 level and COPD vs control (never smokers). Multivariable logistic regression model was adjusted for age, assay plate number, and BMI. Odds of COPD decreased as serum CC16 level (log scale) increased (OR_adj_ = 0.36, 95% CI 0.22–0.57) P_adj_ < 0.0001 (N = 351)

Serum CC16 levels were significantly lower (p = 0.002) among those with COPD (median 8.4 ng/mL) compared to controls (never smokers, median 10.2 ng/mL) (Fig. 1A). Figure 1B depicts the unadjusted and adjusted relationships between CC16 level (log) and COPD, demonstrating that CC16 level (log) was an independent predictor of COPD after controlling for age, assay plate number, and BMI (adjusted odds ratio = 0.36, 95% Confidence Interval [CI] 0.22–0.57, P_adj_ < 0.0001). Another analysis was performed estimating odds of COPD vs all controls and adjusting for smoking status as a confounder in the model along with other covariates (see Additional file 1: Table S3). The strength of CC16 as a predictor approached significance (adjusted odds ratio = 0.71, 95% CI 0.5, 1.0, P_adj_ = 0.06).

**Fig. 2:**
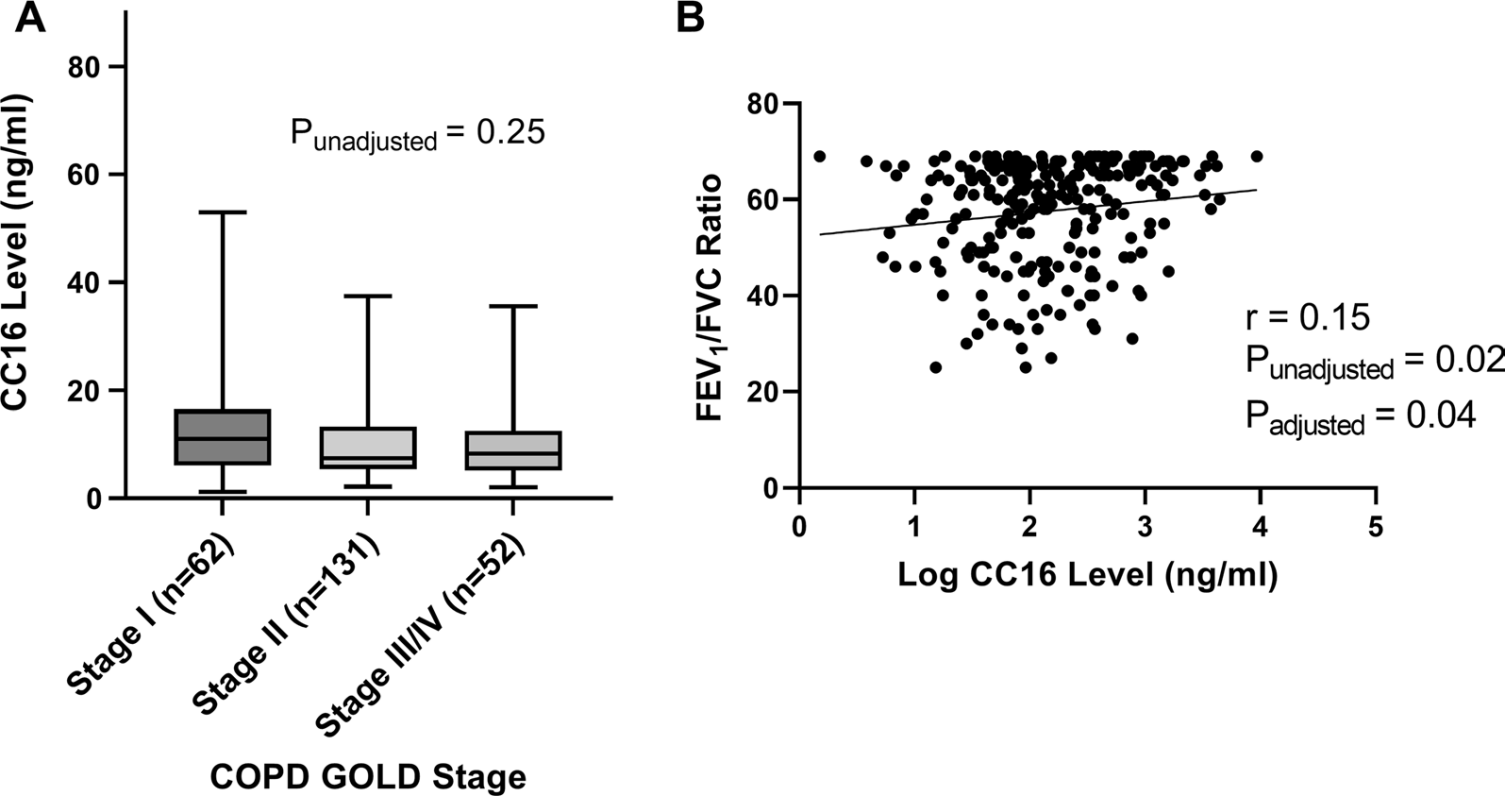
CC16 levels are correlated with FEV_1_/FVC within COPD subjects (N = 245). **A** A box and whisker plot depicts distribution of serum CC16 level across COPD GOLD stages. The median is represented by a solid line and the box represents the interquartile range, whiskers are the minimum and maximum values. There is no difference by stage (ANOVA test using log CC16 level by GOLD stages, p = 0.25). **B** Scatter plot depicts a weak positive correlation between FEV_1_/FVC and log CC16 level in subjects with COPD (r = 0.15, p = 0.02). Adjusted p-value from multiple linear regression model included the following covariates: age, pack-years, smoking status (current, former, never), assay plate number, BMI, and inhaled and systemic steroid use. r = Pearson correlation coefficient

Serum CC16 levels did not vary significantly by COPD GOLD Stage (P = 0.25) (Fig. 2A). Descriptively, CC16 levels were highest in COPD GOLD stage I (11.0 ng/mL) in comparison to stage II (7.4 ng/mL), and stage III/IV (8.3 ng/mL). There was a weak, but statistically significant, positive correlation between CC16 level and the FEV_1_/FVC ratio in COPD (scatter plot, Fig. 2B). This relationship persisted after adjustment for potential confounders (assay plate number, inhaled steroids, systemic steroids, age, pack-years, smoking status, and BMI; see Fig. 2B and Additional file 1: Table S1). Correlations with CC16 levels were not distinguishable from zero for FEV_1_ percent predicted (r = 0.12, p = 0.07) or FVC percent predicted (r = 0.02, p = 0.77). However, in multivariable linear regression modeling, CC16 was associated with FEV_1_ percent predicted (p = 0.03; see Additional file 1: Table S1).

**Fig. 3:**
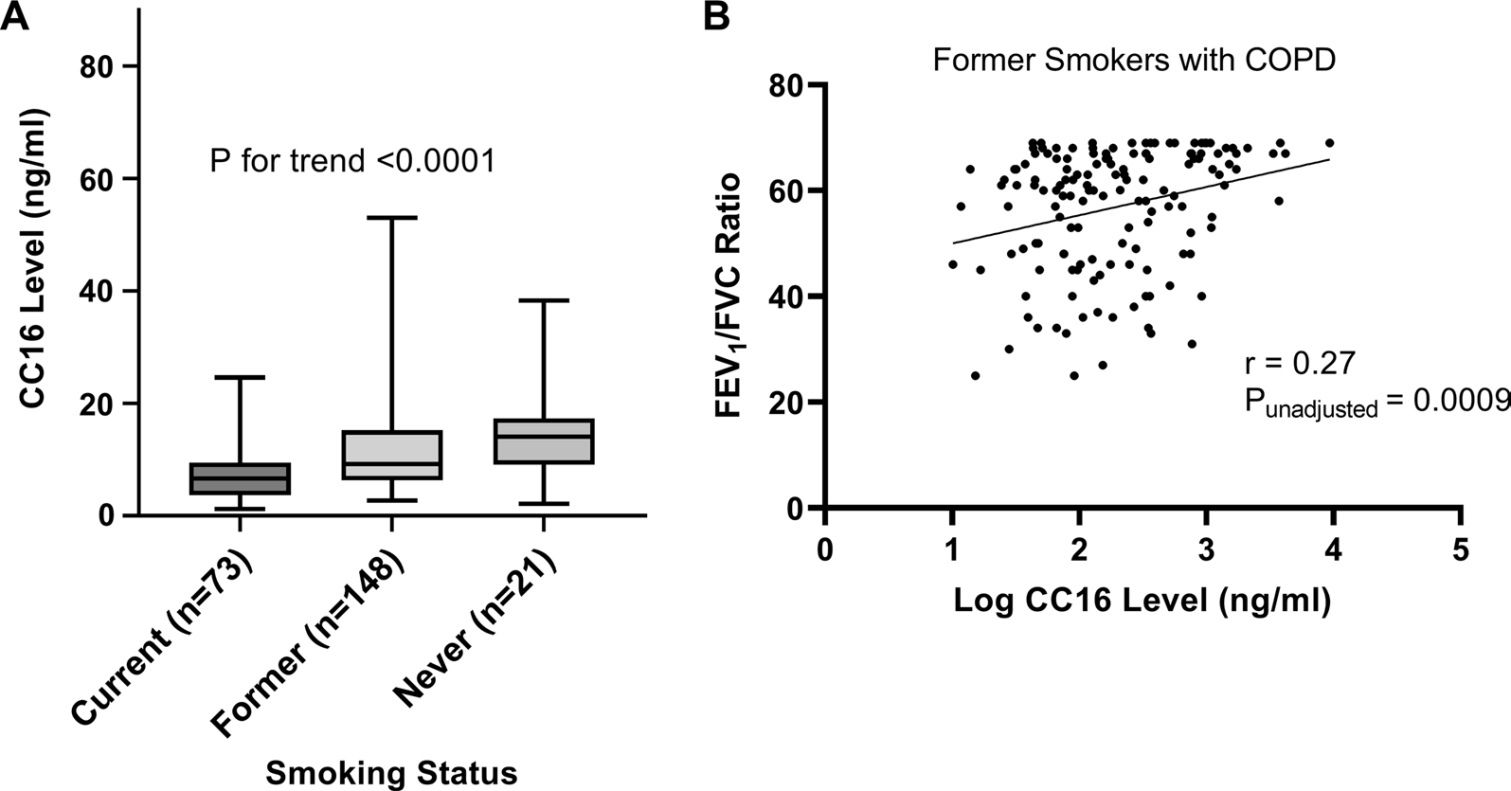
CC16 levels vary by smoking status in COPD (left); CC16 levels are positively correlated with FEV_1_/FVC among former smokers with COPD (right). **A** A box and whisker plot depicts distribution of CC16 level across smoking status. The median is represented by a solid line and the box represents the interquartile range, whiskers are the minimum and maximum. Serum CC16 levels in COPD were highest among never smokers, followed by former and current (N = 245, P for trend < 0.0001). **B** A scatter plot depicts a positive correlation between CC16 level (log) and FEV_1_/FVC among former smokers with COPD (N = 148, p = 0.0009). r = Pearson correlation coefficient

The distribution of serum CC16 level by smoking status among COPD cases is illustrated in Fig. 3A. Serum CC16 levels in COPD were highest among never smokers (14.1 ng/mL), followed by former (9.2 ng/mL), and then current smokers (6.6 ng/mL) (P for trend unadjusted < 0.0001).

**Fig. 4:**
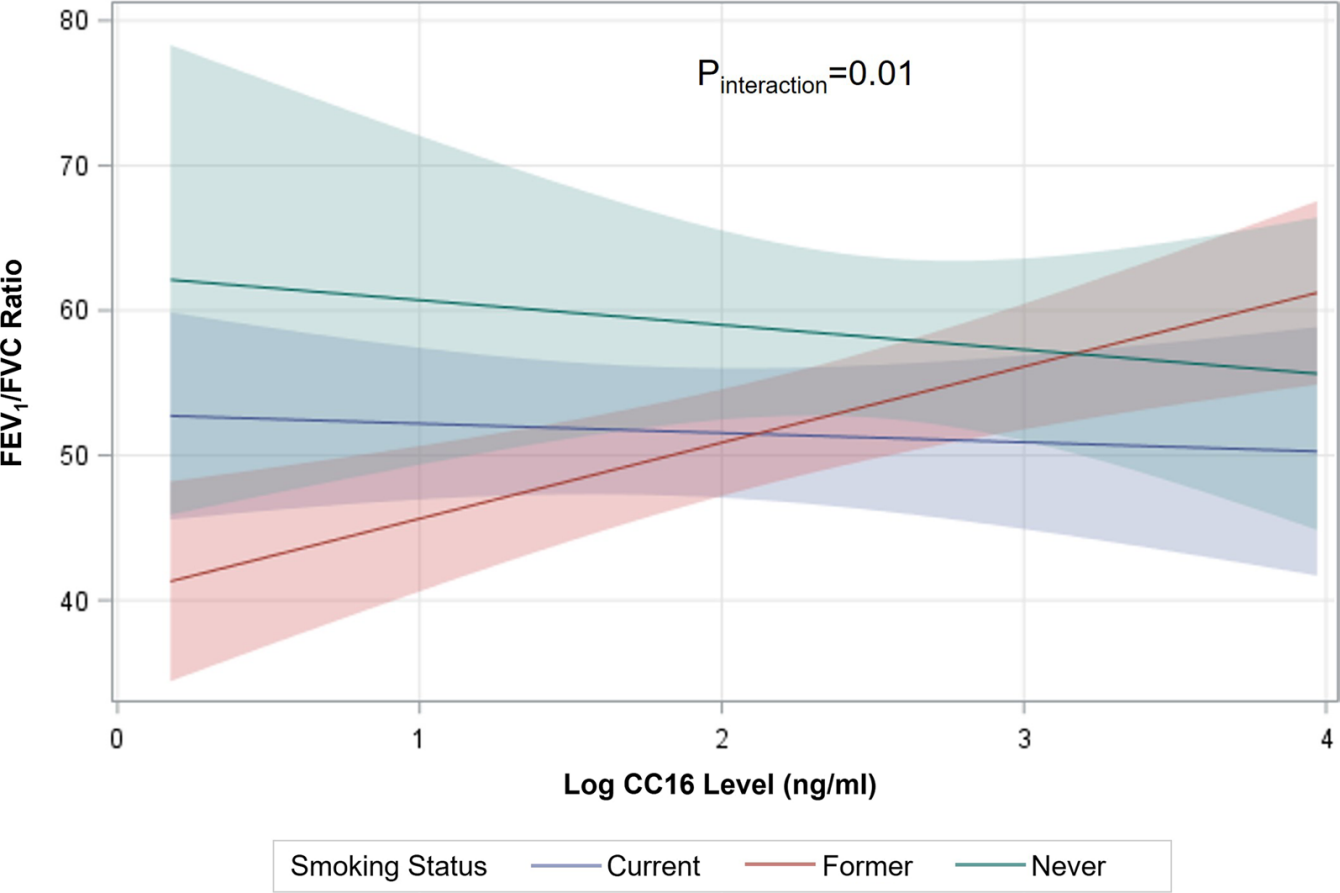
Relationship between FEV_1_/FVC and CC16 level is modified by smoking status among those with COPD (N = 245). The linear regression interaction plot depicts the statistically significant interaction effects between log CC16 level and smoking status on FEV_1_/FVC in COPD (P_interaction_ = 0.01). Presented are regression lines and 95% confidence intervals of the model estimated slopes for each smoking status calculated at fixed values of other variables in the model. For former smokers with COPD, FEV_1_/FVC increased with each unit increase in log CC16 level, while an inverse relationship was noted for never and current smokers with COPD. Along with the dependent variable, the model included the interaction term (CC16 * smoking status), the main effects of smoking status and log CC16 level, age, BMI, pack-years, assay plate number, and inhaled and systemic steroid use

We further investigated relationships between CC16 serum level and lung function measures stratified by smoking status in those with COPD. A positive correlation (r = 0.27, p = 0.0009) was observed between FEV_1_/FVC and serum CC16 levels among former smokers with COPD (Fig. 3B). Although not distinguishable from zero, negative correlations between FEV_1_/FVC ratio and CC16 serum level were found for current (N = 73, r = − 0.14, p = 0.24) and never smokers (N = 21, r = − 0.11, p = 0.62) with COPD. The positive correlation among former smokers and negative correlations among current and never smokers with COPD suggests an interaction. Indeed, the interaction between CC16 levels and smoking status in regards to FEV_1_/FVC ratio in COPD was confirmed in a multivariable linear regression model adjusted for potential confounders age, BMI, pack-years, assay plate number, inhaled and systemic steroid use (p_adj_ = 0.01) (Fig. 4). Additional file 1: Table S2, details the smoking status-stratified model output for the relationship between serum CC16 levels and FEV_1_/FVC ratio. CC16 levels were also correlated with FEV_1_ percent predicted among former smokers with COPD (r = 0.22, p = 0.009). No additional relationships between CC16 level and lung function measures were demonstrated within smoking status groups among those with COPD.

**Fig. 5:**
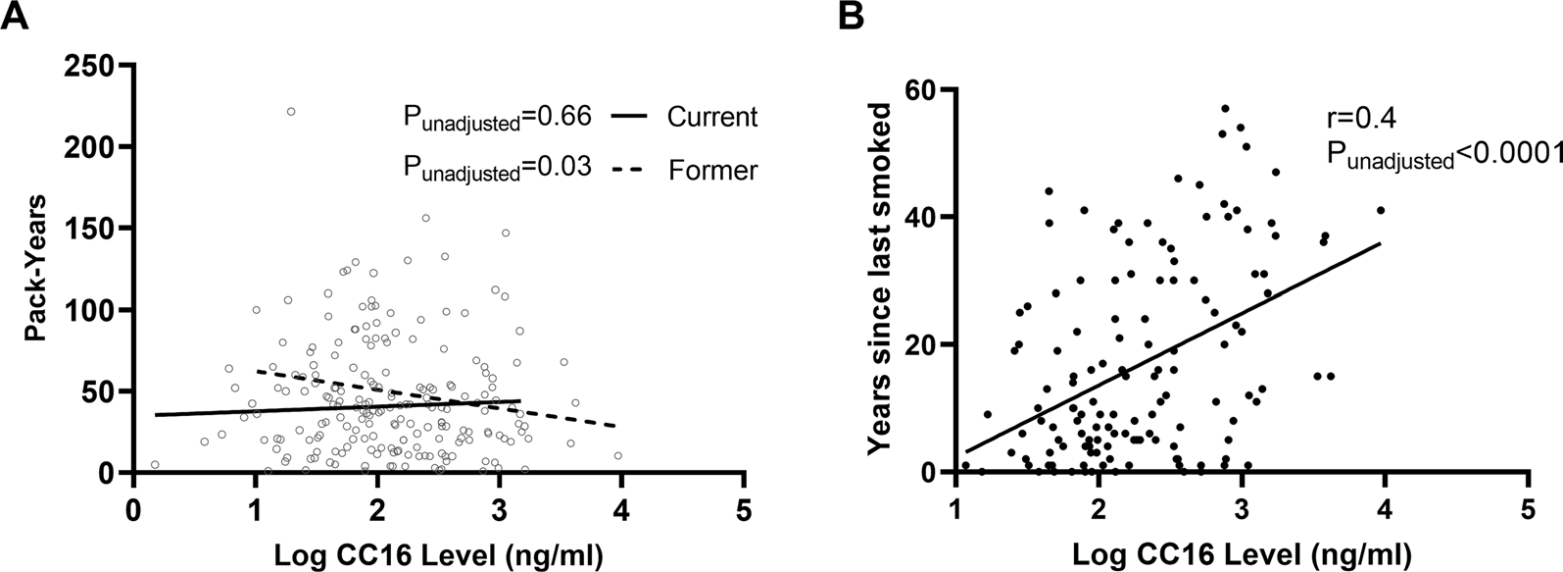
Correlations between CC16 level and pack-years smoked (former and current smokers with COPD) and years since last smoked (former smokers) with COPD. **A** Scatter plot demonstrates correlations between CC16 level (log), and pack years smoked for current (N = 73) and former smokers (N = 148) with COPD. There is no statistically significant correlation between serum CC16 levels and pack-years smoked among current smokers with COPD (p = 0.66). There is a significant inverse relationship between pack-years smoked and CC16 levels among former smokers with COPD (r = − 0.2, p = 0.03); CC16 levels decline as pack-years smoked increase. **B** Scatter plot depicts serum CC16 levels are positively correlated with years since last smoked among former smokers with COPD. r = Pearson correlation coefficient

Next, we explored relationships between CC16 and smoking history in COPD subjects. Among former smoking adults with COPD, there was an inverse relationship between serum CC16 levels and pack-years (p = 0.03), while no correlation was found for current smokers with COPD (Fig. 5A). Furthermore, a positive correlation was found between years since last smoked and CC16 serum level among former smokers with COPD (r = 0.4, p < 0.0001) (Fig. 5B), suggesting a potential recovery of serum CC16 level response over time following smoking cessation. The median time in cessation among former smoking COPD subjects was 11 years, with only 13 (8.8%) reporting a cessation period of less than 1 year.

## Discussion

CC16 is an anti-inflammatory and antioxidant protein in which low serum levels are associated with COPD. In this current study, we confirmed the relationship of low serum CC16 levels in COPD and importantly, demonstrated a novel interaction between smoking status and serum CC16 level on lung function in subjects with COPD. Former smoking, as opposed to current or never smoking, was uniquely associated with lung function as evident by the positive and linear relationship between CC16 serum level and FEV_1_/FVC ratio in former smokers with COPD. Furthermore, serum levels of CC16 positively correlated with duration since quitting smoking, indicating that the reduced serum CC16, presumably induced by cigarette smoke exposure, is reversible, and suggests that CC16 levels may ultimately recover after smoking cessation among COPD subjects.

There has previously been limited evidence of the effects of serum CC16 level on lung function measures among former smokers, analyzed separately from current smokers, within COPD populations. In the Evaluation of COPD Longitudinally to Identify Predictive Surrogate Endpoints (ECLIPSE) COPD cohort, there was a weak correlation between FEV_1_ and CC16 serum level among former smokers (r = 0.11, p =  < 0.001), and a non-statistically significant correlation among current smokers (r = − 0.07, p = 0.069) in a combined sample of subjects with and without COPD (n = 1042 current smokers, n = 1237 former smokers, n = 201 non-smoking controls) [13]. Our findings are consistent with the ECLIPSE findings in that former smokers with COPD had decreased CC16 levels, but in comparison, the strength and direction of the correlations with lung function within COPD were more robust. In contrast, there was no association between CC16 levels and FEV_1_/FVC ratio among current or never smokers with COPD by stratified analysis in our cohort. A potential explanation is that current smoking is known to suppress CC16 expression and associated circulating levels due to the Club cells’ role in detoxifying inhaled xenobiotics [3, 14, 15, 20]. COPD subjects that continue to smoke after diagnosis experience more rapid disease progression (i.e. loss of lung function over time) compared to those that quit smoking. Therefore, CC16 may have an essential role in multiple pathways such as mediating smoke-induced lung injury, regulating inflammatory pathways, and/or structural damage (emphysema). Serum CC16 levels were not associated with FEV_1_/FVC ratio among never smokers with COPD to highlight the relationship of cigarette smoke exposure on epithelial cell CC16 expression [3]. The time course to recovery of CC16 responses or whether CC16 levels ever return to normal homeostasis in COPD subjects with smoking history remains unknown.

Serum CC16 levels were positively correlated with lung function (FEV_1_/FVC ratio) regardless of smoking status in COPD subjects, a finding consistent with previous literature [14]. Namely, Rong and colleagues investigated 98 COPD subjects that were defined as 47% smokers, without delineation of former vs. current smoking status, and demonstrated a positive correlation between CC16 and FEV_1_/FVC among COPD subjects (r = 0.39, p = 0.002) [14]. Whereas we found a relationship between FEV_1_ percent predicted and CC16 level among subjects with COPD in this cross-sectional study, others have suggested that CC16 may be a biomarker of COPD progression (decrease in FEV_1_ over time) [15, 16]. Using longitudinal data collected over three years on 2163 moderate-severe (GOLD stages II, III, IV) COPD subjects within the ECLIPSE study population, it was demonstrated that a one standard deviation decrease in baseline CC16 level was associated with a 4 mL/year greater rate of decline in FEV_1_ [16]. Park et al., confirmed these findings using 9-years of follow-up data from the Lung Health Study participants with COPD (N ~ 4700) reporting an inverse relationship between CC16 level broken into quintiles (quintile 1 lowest CC16 level to quintile 5 highest) and rate of FEV_1_ decline [15]. Compared with the highest CC16 level (quintile 5), the rate of decline in FEV_1_ ranged from 7 mL/year for lowest quintile to 0.88 mL/year in quintile four [15]. In the smoking-stratified analysis, the correlation between CC16 level and FEV_1_ decline was only observed among intermittent quitters as opposed to former smokers or non-smokers [15]. In contrast, Celli and others reported no correlation between CC16 levels and FEV_1_ percent predicted or decline using Study to Understand Mortality and MorbidITy (SUMMIT) randomized controlled trial data on 1673 COPD subjects with increased cardiovascular disease risk [21]. Although, the authors acknowledged CC16 levels were affected by treatment assignment, and relationships were not adjusted for smoking [21]. Due to the heterogenous nature of COPD, variability in CC16 levels, smoking status/history, and changes to lung function over time with aging, medication, or disease progression, additional longitudinal studies in larger COPD cohorts are needed to better understand time-varying and dynamic relationships between CC16 levels, smoking status, and lung function in COPD populations.

CC16 protein is the primary product secreted by Club cells which are present throughout the respiratory epithelium [11]. Club cells play a key role in host defense through the expression of genes involved in immune, anti-protease, anti-bacterial, and physical barrier function [22]. Cigarette smoke exposure directly damages the club cells reducing their abundance, differentiation, biological defense functions, and increases number of mucous-producing cells [3, 8, 11, 22]. As such, smoke-induced injury to club cells and subsequent decreased CC16 may contribute to an increased susceptibility to COPD [12]. In addition to smoke-induced loss of club cells reducing levels of CC16 protein, the gene (SCGB1A1) has been shown to be hypermethylated in the small airway epithelium of healthy smokers compared to non-smokers which may lead to a reduction in gene expression [23]. In our study, serum CC16 levels were inversely associated with odds of COPD, supporting the existing evidence of a protective effect of CC16 on COPD risk [6, 12]. After disease onset, smoking status and history affect CC16 levels. Confirmed in our study and previously described, current smokers had the lowest serum levels of CC16, followed by former, and never smokers with COPD [13–15]. The positive relationship between CC16 and lung function among former smokers with COPD (and not in current or never smokers) is supported by experimental evidence suggesting a role for CC16 in regulating cigarette smoke-induced lung injury in COPD [3, 10, 12].

While lung function in COPD is thought to be influenced by multiple factors including chronic inflammation, ongoing deleterious structural changes, and overproduction of mucus leading to airway obstruction, a complete understanding of mechanisms remains elusive [24]. Experimentally, cigarette-smoke exposed mice lacking CC16 have shown indications of lung injury consistent with COPD pathologies [10, 12]. Utilizing a mouse-model of COPD, mice that received intranasally-administered recombinant CC16 experienced reductions in cigarette-smoked induced pro-inflammatory cytokines (IL-6, IL-8, TNF-α), reduced NF-κB activation, decreases in inflammatory cell infiltration, and reduced alveolar size [10]. Our study supports that the magnitude of smoking history, particularly in former smokers with COPD, is important in CC16 responses because of the inverse relationship found between the number of pack-years smoked and serum CC16 level. Bernard and colleagues also reported that CC16 serum levels decreased by an estimated 15% with each 10-pack-year unit increase in healthy smoking adults [25]. A potential explanation is serum CC16 expression following cessation in COPD subjects may be partially determined by prior smoking intensity (i.e. pack-years). Experimental data to support these notions are currently lacking, but the smoke exposure “dose” could impact amount/degree of damage to respiratory epithelium including the abundance of club cells which secrete CC16 protein. This dose–response relationship suggested by number of pack-years smoked may not be demonstrated in current smokers with COPD due to the ongoing smoke-exposure causing damage to club cells and sustained suppression of CC16 in this sample of current smokers with significant smoking history (average 35 pack-years). Whereas smoke exposure induces long-lasting effects on club cells, this effect, appears to be potentially reversible because we demonstrated increasing levels of CC16 with more time since smoking cessation among former smokers with COPD. This encouraging and novel observation requires further investigation as strategies aimed at increasing effector club cell numbers as well as club cell function that secrete the anti-inflammatory/antioxidant CC16 protein may represent novel approaches to reduce disease burden.

The association of serum CC16 level and COPD GOLD stage is less clear. Rong and colleagues, noted a significantly lower CC16 level in COPD subjects with GOLD stage III or IV compared with I or II [14]. In the ECLIPSE study reported by Lomas and colleagues, there were no differences in the serum CC16 levels by GOLD stage, similar to our findings [13]. Lack of consensus is not surprising given heterogenous COPD study populations (e.g. age, stage, definition of COPD) and variability of CC16 levels across studies. Disease progression and advancing stage is likely influenced by multiple factors that may have varied across prior studies.

Our study has additional limitations. Due to the cross-sectional design of this study, we cannot infer causality. While the finding of an interaction between CC16 and smoking status among COPD subjects was highly statistically significant, we acknowledge the number of never smokers with COPD was small (N = 21) compared to former (N = 148) and current smokers (N = 73) in the analysis likely due to most COPD cases resulting from smoke exposure opposed to other factors that can influence risk (i.e. occupational exposures, genetics). Our cohort was limited to United States veterans in our region that was predominately a male COPD population limiting generalizability. Study participants had at least 2 years of farm exposure, and although we explored but found no evidence of relationships between CC16 and agricultural exposures, it remains possible that agriculture exposures could affect serum CC16 levels, and future studies to include agriculturally unexposed control groups would be warranted to fully understand this relationship. This study did not have a validation cohort which supports the need for evidence of CC16 and lung function outcomes in independent COPD populations. COPD subjects were recruited at a stable state, indicating no history of infection or exacerbation in the previous 3 weeks at the time of enrollment. However, time from last exacerbation was not collected. The number of COPD subjects with advanced disease (i.e. GOLD stage III–IV) in our study population was relatively small (n = 52) potentially prohibiting a complete evaluation of CC16 levels by stage. There may also be differences in smoking history or environmental exposure history from military training and deployments between our sample and other COPD populations. Lastly, we did not measure renal function to evaluate prevalence of chronic kidney disease which can influence CC16 concentrations in serum (i.e. decreased renal clearance of CC16 would elevate serum concentrations).

## Conclusions

Our findings strongly support effect modification by smoking status on the relationship between serum CC16 level and lung function in COPD. We confirmed that CC16 levels are lower in subjects with COPD and current smokers with COPD, and importantly found that with increasing CC16 levels in former smokers with COPD, FEV_1_/FVC ratio correspondingly increased, which was not demonstrated in current or never smokers with COPD. Although limited, the existing mechanistic evidence supports these observations as Club cells, the respiratory cells responsible for secreting CC16 protein, have a role in detoxifying smoke/chemicals, thus rendering them susceptible to damage resulting in reduced numbers and subsequently lower CC16 levels. After cessation, it is plausible Club cells and CC16 levels recover to a certain degree, restoring CC16’s immunoregulatory functions positively influencing lung function among COPD subjects. There is also potential for CC16 recovery with post-smoking cessation that needs to be further investigated as well as its role as a biomarker in COPD. Understanding the role of CC16 in dynamics of COPD and cigarette smoke interplay could also lead to new therapeutic approaches to slow progression, or reverse clinical disease course.

## Supplementary Information

**Additional file 1: Table S1.** Associations between lung function measures, CC16 level, and smoking status among subjects with COPD. **Table S2.** Lung function and CC16 level stratified by smoking status among subjects with COPD (N = 245). **Table S3.** Descriptive and adjusted analysis of CC16 level by COPD vs controls (N = 638).

## Data Availability

Data are not public but may be available upon reasonable request to the corresponding author.

## Abbreviations

BMI: Body mass index
COPD: Chronic obstructive pulmonary disease
CC16: Club cell secretory protein
ELISA: Enzyme-linked immunosorbent assay
FVC: Forced vital capacity
FEV_1_: Forced expiratory volume released in 1 s
FEV_1_/FVC: Ratio of FEV_1_ to FVC
GOLD: Global Initiative for Chronic Obstructive Lung Disease
OR: Odds ratio
