# Supplementary material for: Relationships of serum CC16 levels with smoking status and lung function in COPD
Source: Respir Res. 2022 Sep 16;23:247. doi: 10.1186/s12931-022-02158-8 (PMC9479424; doi:10.1186/s12931-022-02158-8)
Supplement: Supplementary file 1 — Additional file 1: Table S1. Associations between lung function measures, CC16 level, and smoking status among subjects with COPD. Table S2. Lung function and CC16 level stratified by smoking status among subjects with COPD (N = 245). Table S3. Descriptive and adjusted analysis of CC16 level by COPD vs controls (N = 638). [file 12931_2022_2158_MOESM1_ESM.docx]

Relationships of serum CC16 levels with smoking status and lung function in COPD

**Authors**: Gribben KC, Poole JA, Nelson AJ, Farazi E, Wichman CS, Heires AJ, Romberger DJ, LeVan TD

Additional file 1

**Table S1**: Associations between lung function measures, CC16 level, and smoking status among subjects with COPD (N=245)

|  | **FEV_1_/FVC** | | | |  | **FEV_1_ (% predicted)** | | | |  | **FVC (% predicted)** | | | |
| --- | --- | --- | --- | --- | --- | --- | --- | --- | --- | --- | --- | --- | --- | --- |
|  |  | | | |  |  | | | |  |  | | | |
|  | Unadjusted^#^  β  (95%CI) | p | Adjusted^*^  β  (95%CI) | p |  | Unadjusted^#^  β  (95%CI) | p | Adjusted^*^  β  (95%CI) | p |  | Unadjusted^#^ β  (95%CI) | p | Adjusted^*^  β  (95%CI) | p |
|  |  |  |  |  |  |  |  |  |  |  |  |  |  |  |
| **CC16^^^** | 2.4  (0.4, 4.5) | **0.02** | 2.4  (0.2, 4.6) | **0.04** |  | 3.6  (-0.3, 7.5) | 0.07 | 4.5  (0.4, 8.6) | **0.03** |  | 0.5  (-3.1, 4.1) | 0.77 | 3.3  (-0.8, 7.4) | 0.11 |
| **Smoking status** | | **0.01** |  | 0.15 |  |  | **<.0001** |  | **0.0007** |  |  | **<.0001** |  | **0.0003** |
| Never | Ref |  | Ref |  |  | Ref |  | Ref |  |  | Ref |  | Ref |  |
| Former | -7.3  (-12.2, -2.3) |  | -4.7  (-9.4, 0.04) |  |  | -21.2  (-30.2, -12.3) |  | -16.5  (-25.2, -7.7) |  |  | -15.7  (-24.0, -7.4) |  | -14.2  (-22.7, -5.4) |  |
| Current | -5.8  (-11.2, -0.4) |  | -4.4  (-9.4, 0.7) |  |  | -13.6  (-23.5, -3.7) |  | -11.1  (-20.5, -1.7) |  |  | -5.1  (-14.2, 4.0) |  | -5.0  (-14.3, 4.3) |  |

^#^Unadjusted models include the outcome (lung function measure) and predictor variable (Log CC16 level) only.

^*^Adjusted multivariable linear regression models: outcome lung function measure with log CC16 level, age, pack-years, smoking status, assay plate number, BMI, and inhaled and systemic steroid use.

^^^CC16 serum level (ng/ml) log transformed

Statistically significant p-values (p<0.05) are bolded.

**Table S2.** Lung function and CC16 level stratified by smoking status among subjects with COPD (N=245)

|  | FEV_1_/FVC | | FEV_1_ (% predicted) | | FVC (% predicted) | |
| --- | --- | --- | --- | --- | --- | --- |
| Log CC16 Level | β (95%CI) | P_interaction_ | β (95%CI) | P_interaction_ | β (95%CI) | P_interaction_ |
|  |  | **0.01** |  | 0.29 |  | 0.92 |
| **Never** | -1.7 (-9.0, 5.7) |  | -11.0 (-37.5, 15.5) |  | -10.4 (-35.2, 14.5) |  |
| **Former** | 3.8 (0.5, 7.0) |  | 5.5 (-0.4, 11.4) |  | 3.2 (-3.0, 9.4) |  |
| **Current** | 1.0 (-2.4, 4.4) |  | 3.1 (-3.4, 9.6) |  | 3.3 (-2.9, 9.4) |  |

P_interaction_ is the p-value for the interaction term Log CC16 level*smoking status in each lung function model adjusted for age, pack-years, log CC16 level, smoking status, assay plate number, BMI, and inhaled and systemic steroid use. Statistically significant p-values (p<0.05) are bolded.

**Table S3**. Descriptive and adjusted analysis of CC16 level by COPD vs controls (N=638)

|  | **COPD**  **N=245** | **Current/former smoking Controls**  **N=287** | **Never smoked Controls**  **N=106** | **Odds Ratio (95% CI)** | **P_adj_** |
| --- | --- | --- | --- | --- | --- |
| **CC16 level (ng/mL)*** | 8.3 (5.5-13.6) | 9.7 (6.5-14.0) | 10.2 (7.6-15.6) | 0.71  (0.50, 1.00) | 0.06 |

*median value (25^th^ – 75^th^ percentiles)

The multivariable logistic regression model estimated odds of COPD vs controls with log CC16 as predictor adjusted for smoking status, pack-years, assay plate number, age, BMI.
